# Research on the Stationarity of Hexapod Robot Posture Adjustment

**DOI:** 10.3390/s20102859

**Published:** 2020-05-18

**Authors:** Lei Zhang, Fucai Wang, Zenghui Gao, Shuangshuang Gao, Chenghang Li

**Affiliations:** Department of Automation, Ocean University of China, Qingdao 266100, China; wangfucai@stu.ouc.edu.cn (F.W.); gzhly666@stu.ouc.edu.cn (Z.G.); gaoshuangshuang@stu.ouc.edu.cn (S.G.); lichengxing@stu.ouc.edu.cn (C.L.)

**Keywords:** hexapod robot, smooth adjustment, dynamic mapping, attitude space

## Abstract

This paper proposes a smooth adjustment method for the instability problem that occurs during the start and stop of a multi-footed robot during attitude change. First, kinematics analysis is used to establish the mapping relationship between the joint angles of the robot support legs and the body posture. The leg joint angle is a known quantity that can be measured accurately and in real time. Therefore, when the position of the foot end of the support leg is unchanged, a unique set of joint angles can be obtained with the change of body posture at a certain moment. Based on the designed mapping model, the smooth adjustment of the posture can be achieved by the smooth adjustment of the support legs. Second, a constraint index that satisfies the requirements of the robot’s steady adjustment of the robot is given. The S-curve acceleration/deceleration method is used to plan the body’s attitude angle transformation curve, and then the mapping control relationship is used to obtain the control trajectory requirements of the joint to achieve smooth adjustment. In addition, this paper also gives a simple choice and motion control method for the redundancy problem caused by the number of support legs of a multi-footed robot when the attitude is changed. The simulation and prototype experiments verify and analyze the proposed method. The results of comparative experiments show that the posture adjustment method proposed in this paper has continuous acceleration without breakpoints, the speed changes gently during the start and stop phases of the attitude transformation, and there is no sudden change in the entire process, which improves the consistency of the actual values of the attitude planning curve with the target values. The physical prototype experiment shows that the maximum deviation between the actual value of the attitude angular velocity and the target value changes from 62.5% to 5.5%, and the degree of fit increases by 57.0%. Therefore, this study solves the problem of the instability of the fuselage when the robot changes its attitude, and it provides an important reference for the multi-footed robot to improve the terrain adaptability.

## 1. Introduction

Multi-legged robots have the ability to overcome obstacles and adapt to complex terrain, and they can be used as multi-degree-of-freedom operating platforms to complete mobile tasks in rugged areas [1,2]. This is because the robot can adjust its attitude according to the surrounding environment, and the continuous adjustment of the attitude can ensure the efficiency and stability of the robot when completing the mobile task. However, when the robot adjusts its attitude, especially when the robot is working under a large load, the body is prone to shaking due to the discontinuity of the acceleration of the body. The shaking can even cause the robot to tip over. Therefore, it is necessary to study the stability of the robot to solve the problem of body shaking during posture adjustment.

In order to improve the adaptability of hexapod robots on terrain, many scholars have carried out research on posture adjustment from the perspective of robot mechanical structure, force/position control, improved algorithms, and control strategies. Roennau et al. [3] proposed a reactive attitude control method to ensure the stability of the robot under severe rugged terrain by adding an additional rotary joint. Bjelonic et al. [4] studied a new type of hexapod robot, where each leg has five joints, of which two redundant joints improve the robot’s terrain adaptability by controlling the body posture and the direction of the leg relative to the ground. The above improvements to the mechanical structure have improved the adaptability of the robot to higher slopes to a certain extent. Agheli [5] proposed an improved foot force stability margin method, which uses measured contact foot force as a measure of stability, simplifying data and calculation requirements, and making the robot suitable for flat and uneven terrain. Xie et al. [6] proposed a four-legged robot attitude control method that uses the hip-side swing joint moment of the support leg to balance the body’s turnover. Portilla et al. [7] proposed an underwater zero moment point (Uzmp) method to generate the trajectory of the robot’s center of mass. The method stabilizes the balance of the robot before being subjected to any external interference, and successfully realizes the dynamic walking of the legged robot in the underwater environment. KAI-XIAN et al. [8] designed a novel impedance control method for the hydraulic drive unit (HDU) on hydraulically driven legged robots. Experiments show that the HDU with a new impedance control method can solve the problem of robot joint motion stall caused by the disappearance of the load force, and instantaneously switch between position control and force control. This force/position control strategy enables the robot to rely on high-precision sensors to adjust the posture according to the force at the end of the foot. Jemin et al. [9] proposed a neural network control strategy and applied it to the NYmal quadruped robot. Experiments show that NYmal can accurately and energy-efficiently follow high-level body speed commands and can even recover from a fall in a complex configuration. Goldschmidt et al. [10] developed a neural control mechanism for a six-legged robot that can generate basic walking behaviors and enable the robot to effectively perform reactive climbing behaviors. Kim [11] proposed a six-legged robot in which the pressure center control algorithm establishes the robot as an inverted pendulum model with springs and dampers. The sensory feedback controller is used to control the equilibrium position, and the robot can walk stably in free gait. Belter et al. [12] took the Messor robot as their research object, proposed an attitude optimization algorithm based on particle swarm optimization algorithm, and combined body motion planning to achieve body attitude control when crossing obstacles, ultimately proposing a new method for robot pose adjustment from the perspective of improved algorithms. Zhang et al. [13] introduced a complex leg force sensing system and designed a joint torque sensor to implement a force sensing system within the robot control architecture. Belter [14] proposed a new constraint evaluation method for path planning of multi-leg walking robots. Through the application of Gaussian Mixture (GM) to establish the constraint model, with the help of the proposed analytical constraint function, the robot can check the self-collision and the working space within a few microseconds, and effectively plan the robot’s motion. Faigl et al. [15] proposed a self-adaptive control method based on a low-sensor-less robot as a carrier to reduce the stress and torque of the robot structure and servo motor without any additional sensors. Uchida [16], using the attitude control method of the impedance model, taking into account the actuator dynamics, and using the torque of the rotating link and the reaction force of the thigh and calf links as control inputs, established a type I servo system, and designed a linear quadratic integral control system to complete the attitude control of the six-legged robot. Wang [17] proposed a control strategy for the inclined-face walking of the six-legged robot. The robot obtained the rotation matrix through an inertial measurement unit while walking on the inclined face and used the fuzzy controller to adjust the angle of the motor of the support leg to adjust the attitude. Li [18,19] equivalently converted the body motion planning of the additional attitude into independent foot end trajectory planning of each support foot and simplified the inverse solution of the parallel mechanism. The improved control strategy enabled the robot to adjust its pose more intelligently and efficiently. Chen [20,21] established a speed inverse kinematics model of a six-legged walking robot and adopted a proportional–integral–derivative (PID) control strategy for the position and attitude of the robot to realize the closed-loop posture control of the six-legged walking robot. Li et al. [22,23] proposed a method for adaptively adjusting the attitude of a quadruped robot on a slope using a diagonal jogging state. Han et al. [24] developed effective control strategies to allow the robot to pass smoothly over rough terrain. However, these studies on position and posture have not considered the problem of the inertial force of the fuselage when the robot adjusts its attitude, which will cause the robot to shake when the robot is running. Therefore, it is necessary to plan the robot’s posture adjustment to avoid the problem of body shaking and further improve the terrain adaptability of the robot.

This study aimed to solve the problem of robots shaking when adjusting their posture when the robot is fixed at the end point of the foot. First, the stability during posture adjustment was studied. It was found that body shaking during posture adjustment is mainly a result of the shock caused by the sudden change of inertial force due to the discontinuity of acceleration. In this paper, S-curve acceleration and deceleration planning was performed for the attitude discontinuity curve for the problem of acceleration discontinuity, and a posture adjustment method with zero and constant acceleration start and stop was designed. Then, the number of support legs during posture adjustment was judged according to the constraints of the robot’s mechanical structure and terrain, and the legs were divided into support legs and follower legs. From the planned attitude angle curve, the joint angle curve of the support leg was obtained according to the established joint angle-attitude angle mapping model and kinematic model. A high-end polynomial method was used to design the follower legs that did not meet the constraints. Finally, a simulation was performed in the MATLAB (MathWorks, Inc., Natick, MA, USA) environment according to the actual parameters of the robot. Experiments were carried out using a prototype of a six-legged robot developed independently by the laboratory, which verified the correctness of the method. Therefore, this study provided a smooth planning method for the posture adjustment of the six-legged.

## 2. System Overview

The body posture of the six-legged robot is adjusted by the rotation of the joint angles of the support legs. In this section, by establishing the kinematics model and attitude angle-joint angle mapping model of the robot, the relationship between the attitude angle of the body and the joint angles of the support legs is obtained.

### 2.1. Kinematics Model

**Figure 1:**
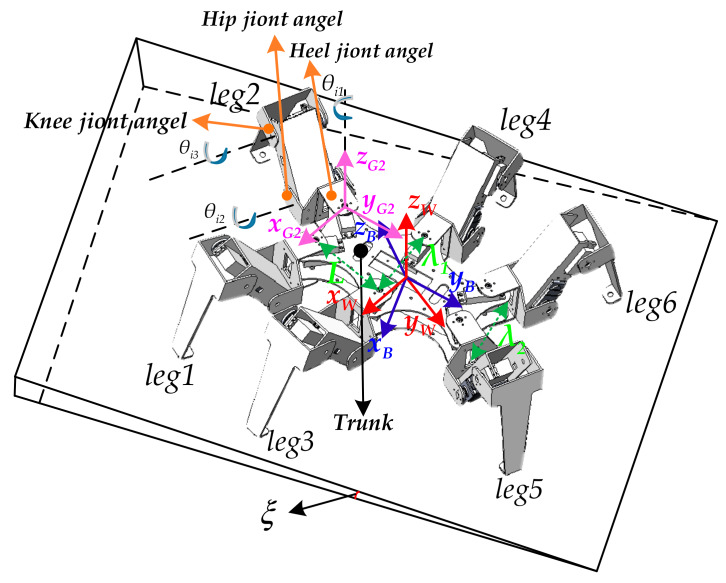
Schematic diagram of the six-legged robot. Here is the distance between the heel joints of *leg3* and *leg4*, $\lambda_{2}$ is the distance between the heel joints of *leg5* and *leg6* (*leg1* and *leg2*), and ξ is the slope of the inclined plane.

In this paper, an insect-like double-triped hexapod robot was taken as the research object. The body was an axisymmetrical hexagon, each leg had three degrees of freedom, the robot’s weight was concentrated in the center of the body, and the center of gravity of the robot and the center of the body were approximately set to coincide. The kinematics model was established based on the simplified structure of the robot shown in Figure 1. The structural parameters are shown in Table 1. The coordinate system was set as follows:

▪ Reference coordinate system ∑W: the origin is located at the center of mass of the robot; $z_{W}$ is opposite to the direction of gravity; the $y_{W}$ axes are pointing directly in front of the robot body in parallel to the horizontal direction; and the $x_{W}$ axes are determined according to the right-hand rule. This coordinate system was used to simplify the description of the body’s posture.
▪ Body coordinate system ∑B: the center of mass of the robot is the origin of the coordinates; the $z_{B}$ axis is perpendicular to the body; the $y_{B}$ axis points directly in front of the body; and the $x_{B}$ axis points right to the robot body.
▪ Heel joint coordinate system $\sum G_{i}$: the origin is the heel joint of the *i*-th leg, and the directions of the coordinate axes are consistent with the coordinate axes of the body coordinate system.

The D-H method was used to obtain the *i*-th leg foot position ${}^{G_{i}}P_{i}$ in the root joint coordinate system ${\sum G}_{i}$,

(1) $$\begin{array}{ll} {}^{G_{i}}P_{i} & ={\left[{}^{G_{i}}x_{i},{}^{G_{i}}y_{i},{}^{G_{i}}z_{i}\right]}^{T}\\ & =\left[\begin{array}{c} {\left(-1\right)}^{i}c_{i1}\left(l_{1}+l_{2}c_{i2}+l_{3}c_{i23}\right)\\ s_{i1}\left(l_{1}+l_{2}c_{i2}+l_{3}c_{i23}\right)\\ l_{2}s_{i2}+l_{3}s_{i23} \end{array}\right] \end{array}$$

where $c_{ij}=cos\theta_{ij}$, $c_{i23}=cos\left(\theta_{i2}+\theta_{i3}\right)$, $s_{ij}=sin\theta_{ij}$, $s_{i23}=\sin\left(\theta_{i2}+\theta_{i3}\right)$, and j=1,2,3; the same below.

According to the one-leg inverse kinematics equation, the joint angle matrix $\theta_{i}={\left[\theta_{i1},\theta_{i2},\theta_{i3}\right]}^{T},\left(i=1,2\ldots 6\right)$ was obtained from the six foot end positions ${}^{G_{i}}P_{i}$, where

(2) $$\begin{array}{l} \theta_{i1}=sgn\left({}^{G_{i}}y_{i}\right)arctan(\frac{\left|{}^{G_{i}}y_{i}\right|}{\left|{}^{G_{i}}x_{i}\right|})\\ \theta_{i2}=\left|arccos\frac{l_{2}^{2}+x_{i}^{\prime }{}^{2}+{}^{G_{i}}z_{i}^{2}-l_{3}^{2}}{2l_{2}\sqrt{x_{i}^{\prime }{}^{2}+{}^{G_{i}}z_{i}^{2}}}\right|-arctan\frac{\left|{}^{G_{i}}z_{i}\right|}{\left|x_{i}^{\prime }\right|}\\ \theta_{i3}=\left|arccos\frac{l_{2}^{2}+l_{3}^{2}-\left(x_{i}^{\prime }{}^{2}+{}^{G_{i}}z_{i}^{2}\right)}{2l_{2}l_{3}}\right|-\pi \end{array}$$

where $x_{i}^{\prime }=\frac{{}^{G_{i}}x_{i}}{cos\theta_{i1}}-sgn\left({}^{G_{i}}x_{i}\right)l_{1}$.

When the foot is moving, the transformation relationship between the foot end speed of the *i*-th leg of the robot and the angular velocity of the joint is

(3) $${\dot{\theta }}_{i}={\left[{\dot{\theta }}_{i1},{\dot{\theta }}_{i2},{\dot{\theta }}_{i3}\right]}^{T}=\left(J^{-1}{}_{i}\right){}^{G_{i}}{\dot{P}}_{i}$$

$J_{i}$ is the Jacobian matrix:

(4) $$J_{i}=\left[\begin{array}{ccc} J_{11} & J_{12} & J_{13}\\ J_{21} & J_{22} & J_{23}\\ J_{31} & J_{32} & J_{33} \end{array}\right]$$

where $J_{11}=-s_{i1}\left(l_{1}+l_{2}c_{i2}+l_{3}c_{i23}\right)$, $J_{12}=-c_{i1}\left(l_{2}s_{i2}+l_{3}s_{i23}\right)$, $J_{13}=-l_{3}c_{i1}s_{i23}$, $J_{21}=c_{i1}\left(l_{1}+l_{2}c_{i2}+l_{3}c_{i23}\right)$, $J_{22}=-s_{i1}\left(l_{2}s_{i2}+l_{3}s_{i23}\right)$, $J_{23}=-l_{3}s_{i1}s_{i23}$, $J_{31}=0$, $J_{32}=l_{2}c_{i2}+l_{3}c_{i23}$, and $J_{33}=l_{3}c_{i23}$.

By establishing the forward and inverse kinematics model, the corresponding relationship between the leg joint angle and the foot end position was obtained. The velocity Jacobian matrix of (3) was used to prepare for the later establishment of a model of attitude angular velocity and joint angular velocity mapping.

### 2.2. Pose-Joint Angle Mapping Model

**Figure 2:**
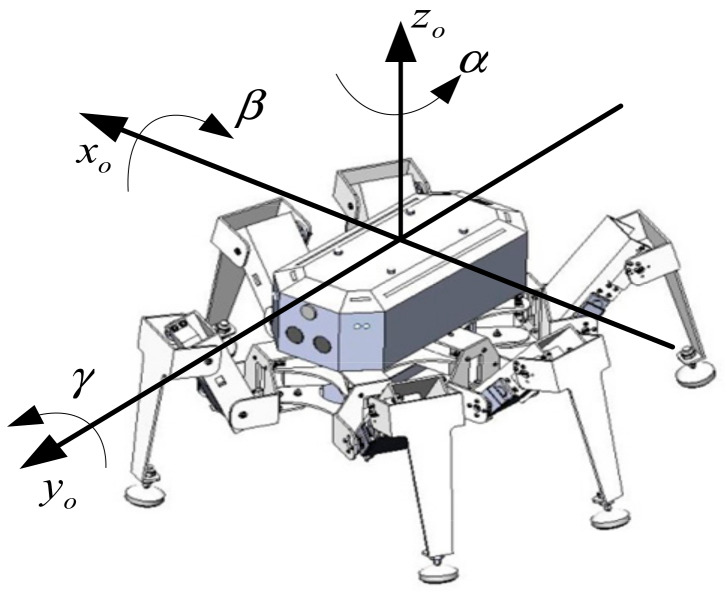
Robot body motion posture description.

As shown in Figure 2, the attitude angle matrix $E={\left[\alpha ,\beta ,\gamma \right]}^{T}$ of the six-legged robot was defined, where α,β, and γ represent the yaw angle, pitch angle, and roll angle of the robot posture, respectively.

Relative to the reference coordinate system, the angular relationship of the rotation of each axis of the body coordinate system can be described by the rotation matrix ${}^{W}R_{B}$,

(5) $$\begin{array}{ll} {}^{W}R_{B} & =Rot\left(z_{o},\alpha \right)Rot\left(x_{o},\beta \right)Rot\left(y_{o},\gamma \right)\\ & =\left[\begin{array}{ccc} c_{\alpha }c_{\beta } & -s_{\alpha }c_{\gamma }+c_{\alpha }s_{\beta }s_{\gamma } & s_{\alpha }s_{\gamma }+c_{\alpha }s_{\beta }c_{\gamma }\\ s_{\alpha }c_{\beta } & c_{\alpha }c_{\gamma }+s_{\alpha }s_{\beta }s_{\gamma } & -c_{\alpha }s_{\gamma }+s_{\alpha }s_{\beta }c_{\gamma }\\ -s_{\beta } & c_{\beta }s_{\gamma } & c_{\beta }c_{\gamma } \end{array}\right] \end{array}$$

The relationship between the coordinates of the foot endpoint in the heel joint coordinate system and the coordinates in the body coordinate system can be expressed as

(6) $${}^{B}P_{i}={}^{B}P_{G_{i}}+{}^{G_{i}}P_{i}$$

where ${}^{B}P_{i}$ is the position of the heel joint in ∑B, which is determined by the structure of the body:

(7) $$\begin{array}{cc} {}^{B}P{}_{Gi}= & \left[\begin{array}{cccccc} {}^{B}P{}_{G1} & {}^{B}P{}_{G2} & {}^{B}P{}_{G3} & {}^{B}P{}_{G4} & {}^{B}P{}_{G5} & {}^{B}P{}_{G6} \end{array}\right]\\ & =\left[\begin{array}{cccccc} -\frac{\lambda_{1}}{2} & \frac{\lambda_{1}}{2} & -\frac{\lambda_{2}}{2} & \frac{\lambda_{2}}{2} & -\frac{\lambda_{1}}{2} & \frac{\lambda_{1}}{2}\\ L & L & 0 & 0 & -L & -L\\ 0 & 0 & 0 & 0 & 0 & 0 \end{array}\right] \end{array}$$

The relationship between the coordinates of the foot endpoint in the body coordinate system and the coordinates in the reference coordinate system can be expressed as

(8) $${}^{W}P_{i}={}^{W}R_{B}{}^{B}P_{i}$$

where ${}^{B}P_{i}$ is the position of the leg end of the support leg in the body coordinate system; ${}^{W}P_{i}$ is the position of the leg end of the support leg in the reference coordinate system; and ${}^{W}R_{B}$ is the rotation matrix of the body coordinate system relative to the reference coordinate system.

Since ${}^{W}R_{B}$ is an orthogonal matrix, ${}^{W}R_{B}{}^{-1}={}^{W}R_{B}{}^{T}$. From Equations (6) and (8), the relationship between the position of the foot end in the heel joint coordinate system and the attitude angle can be expressed as

(9) $${}^{G_{i}}P_{i}={}^{W}R_{B}{}^{T}{}^{W}P_{i}-{}^{B}P_{Gi}$$

Therefore, when the position of the support foot is fixed, the mapping relationship between the position of each support leg in the coordinate system of the heel joint and the attitude angle can be established by Equation (9). According to this position, the joint angle under the current body attitude angle can be further obtained from inverse kinematics. Equations (2) and (9) provide a mapping model for the attitude angle of the body and the joint angle of the support leg when the end points of the support leg and foot are fixed.

## 3. Smooth Adjustment Method of Robot Posture

In order to enable the robot to perform a smooth posture adjustment, it is necessary to determine a smooth adjustment target and plan an attitude angle curve. From the planned attitude angle curve, according to the attitude angle-joint angle mapping model and kinematic model, the joint angle change curve of the support leg at the fixed foot position can be obtained.

### 3.1. Body Steady Adjustment Target

In order to achieve the goal of stability with no shaking of the body when adjusting the attitude, the constraint indicators that the six-legged robot should meet were established as follows.

1. The attitude angle should not change too quickly at the beginning and end of the adjustment;
2. The angular velocity of the attitude is zero at the beginning and end of the adjustment cycle, and the curve should be continuous without breakpoints;
3. The angular acceleration of the attitude is zero at the beginning and end of the adjustment period, and the curve is continuous without breakpoints;
4. During the adjustment of attitude angle, the support leg joint angle must be within the rotation angle range, and the joint angular velocity cannot exceed the allowable limit value.

The above goals are equivalent to the following constraints:

(10) $$\left\{\begin{array}{c} E(0)=E_{f},E(T)=E_{a}\\ \dot{E}(0)=0,\dot{E}(T)=0\\ \ddot{E}(0)=0,\ddot{E}(T)=0\\ \theta_{ij}\in \left[\theta_{ijmin,}\theta_{ijmax}\right],v_{ij}\leq V_{e},i\in [1,6];j\in [1,3]\\ \underset{t->a^{+}}{lim}\dot{E}(t)=\underset{t->a^{-}}{lim}\dot{E}(t)=\dot{E}(a),a\in [0,T]\\ \underset{t->a^{+}}{lim}\ddot{E}(t)=\underset{t->a^{-}}{lim}\ddot{E}(t)=\ddot{E}(a),a\in [0,T] \end{array}\right.$$

In Equation (10), $E_{f}$ is the initial attitude angle; $E_{a}$ is the target attitude angle; $E\left(t\right)$ is the attitude angle curve; $\dot{E}\left(t\right)$ is the attitude angular velocity curve; $\ddot{E}\left(t\right)$ is the attitude angular acceleration curve; $\theta_{ij}$ is the angle of the *j*-th joint of the *i*-th leg; $\theta_{ijmin}$ is the minimum angular value of the joint; $\theta_{ijmax}$ is the maximum angular value of the joint; $v_{ij}$ is the angular velocity of the joint; $V_{e}$ is the limit of the angular velocity of the joint; and T is the adjustment period.

### 3.2. Planning Method Based on S-Curve Acceleration/Deceleration

According to the D’Alembert’s principle of the particle, the main force acting on the particle, the binding force, and the imaginary inertia force form a balanced force system in form. The equivalent inertial force at the real-time center of mass of the robot can be expressed as

(11) $$F_{I}=\left(m_{B}+m_{l}\right)\ddot{E}\left(t\right)+\sum_{i=1}^{6}m_{legi}{}^{W}{\ddot{P}}_{\mathrm{legi}}$$

In the formula, $m_{B}$, $m_{l}$ are the mass of the robot body and the load carried by it, and $m_{legi}$ is the mass of the *i*-th leg of the robot. ${}^{W}{\ddot{P}}_{\mathrm{legi}}$ is the acceleration matrix of the *i*-th leg.

Because the mass of the torso of the robot is large and the mass of the legs is negligible, the main factor affecting the inertial force of the robot is the angular acceleration of the posture of the body. Reasonable planning attitude angle curve during robot posture adjustment can reduce the influence of inertial force.

In order to clearly describe the movement of $E\left(t\right)$ in its value range, the amount of calculation when the initial and target angles and attitude angles were changed was simplified so that $\tau =\frac{t}{T}$. Hence, the attitude angle change curve could be described as

(12) $$\left\{\begin{array}{c} E\left(t\right)=E_{f}+\left(E_{a}-E_{f}\right)s\left(\tau \right)\\ \dot{E}\left(t\right)=\frac{1}{T}\left(E_{a}-E_{f}\right)s^{\prime }\left(\tau \right)\\ \ddot{E}\left(t\right)=\frac{1}{T^{2}}\left(E_{a}-E_{f}\right)s^{\prime \prime }\left(\tau \right) \end{array}\right.$$

Each element value in $E\left(t\right)$ was not greater than the corresponding element value in $E_{a}$. Hence, $0\leq s\left(\tau \right)\leq 1$, where $s\left(\tau \right)$ is the proportionality factor and independent variable of the angle change, and it describes the change process of the angle. The design of the $s\left(\tau \right)$ function can realize the planning of the attitude angle.

Based on the constraints of multiple indicators above, a five-stage “S” curve acceleration and deceleration planning method was used for interpolation. The curve was divided into five sections: acceleration, acceleration, deceleration, uniform speed, deceleration, and deceleration. Except for the uniform speed section, the other four stages were symmetrical. The slopes of the four shift sections were A, the time of the four sections was $T_{a}$, and the acceleration and deceleration section displacements were $L_{1}$ and $L_{2}$.

Then

(13) $$\left\{\begin{array}{l} L_{1}=\frac{1}{6}AT_{a}^{3}\\ L_{2}=\frac{5}{6}AT_{a}^{3}\\ T=4T_{a}+\frac{1-2L_{1}-2L_{2}}{AT_{a}^{2}} \end{array}\right.$$

Then the acceleration function of s(τ) is

(14) $$s{\left(\tau \right)}^{\prime \prime }=\left\{\begin{array}{l} A\tau ,(0\leq \tau \leq T_{a})\\ -A(\tau -2T_{a}),(T_{a}\leq \tau \leq 2T_{a})\\ 0,(2T_{a}\leq \tau \leq T-2T_{a})\\ -A\;[\tau -(T-2T_{a})],(T-2T_{a}\leq \tau \leq T-T_{a})\\ A\;(\tau -T),(T-T_{a}\leq \tau \leq T) \end{array}\right.$$

The velocity function of s(τ) can be obtained by integrating the acceleration,

(15) $$s{\left(\tau \right)}^{\prime }=\left\{\begin{array}{l} \frac{1}{2}A\tau^{2},(0\leq \tau \leq T_{a})\\ -\frac{1}{2}A(\tau -2T_{a}{)}^{2}+AT_{a}^{2},(T_{a}\leq \tau \leq 2T_{a})\\ AT_{a}^{2},(2T_{a}\leq \tau \leq T-2T_{a})\\ -\frac{1}{2}A(\tau -T+2T_{a}{)}^{2}+AT_{a}^{2},(T-2T_{a}\leq \tau \leq T-T_{a})\\ \frac{1}{2}A(\tau -T{)}^{2},(T-T_{a}\leq \tau \leq T) \end{array}\right.$$

The s(τ) function can be obtained by integrating the speed,

(16) $$s(\tau )=\left\{\begin{array}{l} \frac{1}{6}A\tau^{3},(0\leq \tau \leq T_{a})\\ -\frac{1}{6}A(\tau -2T_{a}{)}^{3}+AT_{a}^{2}\tau -AT_{a}^{3},(T_{a}\leq \tau \leq 2T_{a})\\ AT_{a}^{2}\tau -AT_{a}^{3},(2T_{a}\leq \tau \leq T-2T_{a})\\ -\frac{1}{6}A(\tau -T+2T_{a}{)}^{3}+AT_{a}^{2}\tau -AT_{a}^{2},(T-2T_{a}\leq \tau \leq T-T_{a})\\ \frac{1}{6}A(\tau -T{)}^{3}-2AT_{a}^{3}+AT_{a}^{2}T,(T-T_{a}\leq \tau \leq T) \end{array}\right.$$

In this section, according to the goal of smooth posture adjustment, an acceleration and deceleration planning method with zero acceleration starting and ending and continuous changes was designed. The robot can realize smooth posture adjustment according to this method, which is called the smooth adjustment method. The next section will analyze the preparations before posture adjustment.

## 4. Hexapod Robot Support Leg Judgement

Because the six-legged robot may adjust the position of a certain leg of the support leg during posture adjustment, it is necessary to analyze and classify all support legs before posture adjustment and then perform attitude angle planning.

### 4.1. Support Leg Discrimination Algorithm

All supporting legs involved in the posture adjustment process need to satisfy the joint Angle constraint. During the adjustment, the robot needs to be stable. The dynamic stability margin is chosen to measure the stability of the robot due to the influence of adding speed in the robot planning process. The pressure center method was selected as the criterion for dynamic stability [25,26]. $S_{cop}$ is defined as the projection of the center of mass to the support surface along with the external force. If the projection point is in the support polygon, that is $S_{cop}>0$,the robot is stable.

Selection process of support legs

Set the initial flags of all support legs to “1.” The joint angle obtained by using the joint angle sensor can be obtained according to Equations (1), (6), and (8) to obtain the position of the supporting leg and foot end in the reference coordinate system. Under the premise of fixed foot position, Equations (2) and (9) can establish a mapping model of attitude angle-joint angle. From the target posture $E_{a}$, the three joint angles of each support leg are obtained by Equations (2) and (9), and the joint angle is compared with the joint angle constraint range. If any joint angle of a support leg exceeds the constraint range, the leg flag is immediately set to “0” as a follower leg for planning.

The supporting polygon formed by all the legs satisfying the supporting angle constraint, and then the dynamic stability margin is judged. If the entire adjustment process satisfies the dynamic stability constraint, all legs marked “1” participate in the attitude angle adjustment process; otherwise, the robot is unstable and needs to reset $E_{a}$ until the joint angle constraint and stability constraint index are both satisfied. The process ends.

Support leg judgement is shown in Algorithm 1.



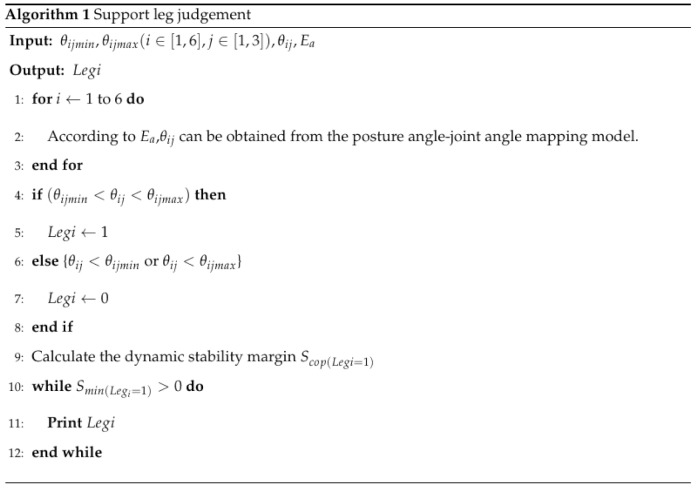



### 4.2. Support Leg Planning

For the leg that passes Algorithm 1, the flag is still “1” as the support leg is involved in posture adjustment. On the premise of the fixed position of the foot end of the support leg, according to the $E\left(t\right)$ curve planned above, the joint angle $\theta_{ij}$ corresponding to the attitude angle at each time t is obtained, so that the support leg joint angle corresponds to the attitude angle planning curve at each moment in the change curve $\theta_{ij}\left(t\right)$.

When the foot of the support leg does not slide, (8) is used to derive the time to obtain the following expression:

(17) $$0={}^{W}{\dot{R}}_{B}{}^{B}P_{i}+{}^{W}R_{B}{}^{B}{\dot{P}}_{i}$$

Because of this relationship,

(18) $${}^{W}{\dot{R}}_{B}=S\left(\omega \right){}^{W}R_{B}$$

where $S\left(\omega \right)$ is the antisymmetric matrix of the attitude angular velocity vector,

(19) $$S\left(\omega \right)=\left[\begin{array}{ccc} 0 & -\omega_{\alpha } & \omega_{\beta }\\ \omega_{\alpha } & 0 & -\omega_{\gamma }\\ -\omega_{\beta } & \omega_{\gamma } & 0 \end{array}\right]$$

Equation (17) can be written as

(20) $$0=S\left(\omega \right){}^{W}R_{B}{}^{B}P_{i}+{}^{W}R_{B}{}^{B}{\dot{P}}_{i}$$

Thus, the speed of the foot end point in the body coordinate system can be expressed as

(21) $${}^{B}{\dot{P}}_{i}=-{}^{W}R_{B}{}^{T}S\left(\omega \right){}^{W}R_{B}{}^{B}P_{i}$$

The derivative of (6) can be obtained, ${}^{B}{\dot{P}}_{i}={}^{G_{i}}{\dot{P}}_{i}$, and the simultaneous Equation (3) can obtain the change curve of the angular velocity of the support leg joint when the attitude angle changes.

(22) $${\dot{\theta }}_{ij}(t)=-J_{i}{}^{-1}{}^{W}R_{B}{}^{T}S\left(\omega \right){}^{W}R_{B}{}^{B}P_{i}$$

### 4.3. Planning of Follower Leg

The follower leg is a support leg that cannot cooperate with the current posture adjustment range. Therefore, it is necessary to plan a new footing point for the follower leg and move it to a new position following the posture adjustment cycle, so as to ensure that the leg will not affect the posture adjustment process due to the problem of joint angle limitation. This avoids the problem that the follower legs hang in the air or affect the movement of the body during posture adjustment.

The plan is presented in this section by taking the supporting surface as a plane as an example. The follower leg and foot end point is from the initial point ${}^{W}P_{f}$ to the target landing point ${}^{W}P_{a}$. The planning of follower leg period *T* and the posture adjustment period *T* remain synchronized.

In order to simplify the calculation, this article uses the octahedron as the reachable area of the foot. The intersection of an octahedron and a plane or slope with a known inclination produces an effective foot reachable area [27,28]. The boundaries of this area are $x_{min}$, $x_{max}$, $y_{min}$, $y_{max}$, $z_{min}$, and $z_{max}$. At the same time, the center position of the effective reachable area is selected as the new foothold ${}^{W}P_{a}$ of the following leg. Then, the expected end point coordinate ${}^{W}P_{a}\left(t\right)=\left[x_{a},y_{a},z_{a}\right]$ is

(23) $$\left\{\begin{array}{c} x_{a}=\frac{x_{min}+x_{max}}{2}\\ y_{a}=\frac{y_{min}+y_{max}}{2}\\ z_{a}=\frac{z_{min}+z_{max}}{2} \end{array}\right.$$

The trajectory of foot ${}^{W}P_{i}(t)$ must satisfy the constraint condition of (24):

(24) $$\left\{\begin{array}{c} {}^{W}P_{i}(0)={}^{W}P_{f},{}^{W}{\dot{P}}_{i}(0)=0,{}^{W}{\ddot{P}}_{i}(0)=0\\ {}^{W}P_{i}(T)={}^{W}P_{a},{}^{W}{\dot{P}}_{i}(T)=0,{}^{W}{\ddot{P}}_{i}(T)=0 \end{array}\right.$$

In Equation (24), ${}^{W}P_{f}$ is the initial foot end position of the follower leg; ${}^{W}P_{a}$ is the target foot end position of the follower leg; and ${}^{W}P_{i}\left(t\right)$,${}^{W}{\dot{P}}_{f}\left(t\right)$, and ${}^{W}{\ddot{P}}_{i}\left(t\right)$ are the position, velocity, and acceleration curves of the foot end in the reference coordinate system.

According to the constraint condition of (24), the fitting of the end-point curve by the high-order polynomial method can be described as

(25) $${}^{W}P_{i}(t)=a_{0}+a_{1}t+a_{2}t^{2}+a_{3}t^{3}+a_{4}t^{4}+a_{5}t^{5}$$

Therefore, the trajectory of the follower leg ${}^{W}P_{f}(t)={\left[\begin{array}{ccc} {}^{W}x(t), & {}^{W}y(t), & {}^{W}z(t) \end{array}\right]}^{T}$ in the reference coordinate system can be described as

(26) $$\left\{\begin{array}{c} {}^{W}x(t)={}^{W}x(0)+\frac{10({}^{W}x(T)-{}^{W}x(0))}{T^{3}}+\frac{15({}^{W}x(0)-{}^{W}x(T))}{T^{4}}+\frac{6({}^{W}x(T)-{}^{W}x(0))}{T^{5}}\\ {}^{W}y(t)={}^{W}y(0)+\frac{10({}^{W}y(T)-{}^{W}y(0))}{T^{3}}+\frac{15({}^{W}y(0)-{}^{W}y(T))}{T^{4}}+\frac{6({}^{W}y(T)-{}^{W}y(0))}{T^{5}}\\ {}^{W}z(t)={}^{W}z(0)+\frac{10({}^{W}z(T)-{}^{W}z(0))}{T^{3}}+\frac{15({}^{W}z(0)-{}^{W}z(T))}{T^{4}}+\frac{6({}^{W}z(T)-{}^{W}z(0))}{T^{5}} \end{array}\right.$$

In Equation (26), ${}^{W}x(t)$, ${}^{W}y(t)$, and ${}^{W}z(t)$ are the change curves of the end points of the foot in the direction of the three coordinate axes in the reference coordinate system.

When the initial and target positions of the follower legs and feet are known and the planning period T is known, the foot-end planning curve can be obtained according to Equation (26).

In this section, motion planning was performed for the support leg and the follower leg when the posture was adjusted smoothly. In the next section, a simulation experiment will be carried out on the body posture smooth adjustment strategy.

## 5. Simulation

First, Algorithm 1 is used to judge each supporting leg of the robot. For the supporting legs that satisfy Algorithm 1, the angle sensor is used to collect the joint angle, and the position of the foot end in the reference coordinate system is calculated. Then, under the condition that the foot position of the supporting leg is fixed, the attitude angle trajectory is planned according to the known initial posture and target posture. Finally, according to the planned attitude angle curve, the attitude angle-joint angle mapping model is used to obtain the change curve of the support leg joint angle. Analyze and evaluate the joint angle change curve to verify the feasibility of this attitude adjustment method.

The simulation environment of the robot was terrain with a slope of 5. The support legs were *leg1*, *leg2*, *leg3*, and *leg6*, whereas *leg4* and *leg5* did not touch the ground. The specific parameter values of the hexapod robot prototype are shown in Table 2. The experimental parameters are shown in Table 3. On the premise that the foot ends of the support legs were not sliding relative to the ground, the posture of the body was changed from the initial attitude angle $E_{f}={\left[0^{{}^{\circ}},5^{{}^{\circ}},0^{{}^{\circ}}\right]}^{T}$ to the target attitude angle $E_{a}={\left[5^{{}^{\circ}},18^{{}^{\circ}},10^{{}^{\circ}}\right]}^{T}$.

According to the judgment of Algorithm 1, it was determined that the knee angle of *leg1* was outside the upper and lower limits of the joint angle, the position of the leg state was set to “0,” and it moved as a “follower leg.” However, the joint angles of *leg2*, *leg3*, and *leg6* were within the constraint range. All joint angles were within the constraint range, the whole process satisfies the dynamic stability margin, so it served as a support leg.

The simulation parameters were set, and the slopes of the four shift sections were smoothly adjusted to $A=8^{{}^{\circ}}/s^{3}$ and $T_{a}=0.3\;s$. From the planned attitude angle curve $E\left(t\right)$ and attitude angular velocity curve $\dot{E}\left(t\right)$, according to Equations (2), (9), and (22), the support leg joint angle curve $\theta_{ij}\left(t\right)$ and the angular velocity curve ${\dot{\theta }}_{ij}\left(t\right)$ were obtained. Because the extreme value of the continuous derivative function can always be obtained at the point where its derivative function is zero, the joint angular velocity curve can obtain the extreme value at $t=2T_{a}$ or $t=T-2T_{a}$. The speed extremes of the three joint angles of the support leg are presented as $V_{e}$ in Table 2. For each joint, the corresponding adjustment period $T_{ij}$ was obtained, and the largest of all $T_{ij}$ was taken as the adjustment period. This adjustment period T was not only the time to satisfy all joint angular velocity constraints but also the fastest time to achieve this adjustment process. After calculating the knee joint corresponding to *leg3*, the adjustment time required was the largest, so the adjustment period was $T=T_{33}=1.98\;s$.

**Figure 3:**
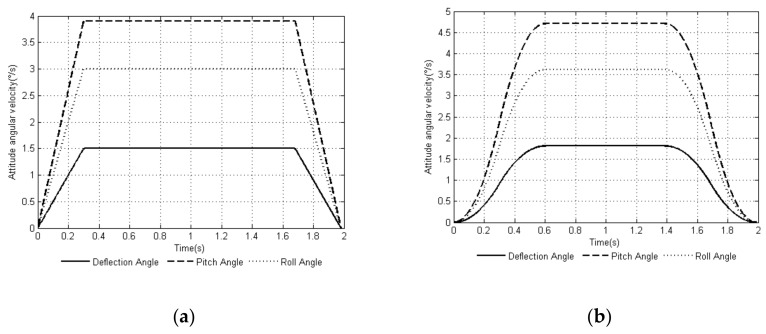
Time-curve of attitude angular velocity based on two adjustment methods: (**a**) uniform speed adjustment method and (**b**) smooth adjustment method.

**Figure 4:**
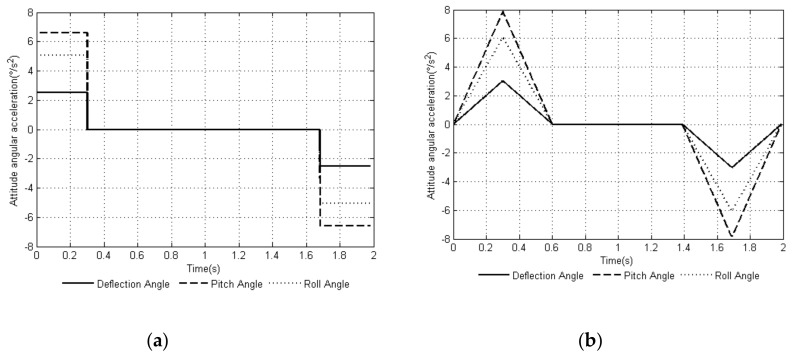
Time-curve of attitude angular acceleration based on two adjustment methods: (**a**) uniform speed adjustment method and (**b**) smooth adjustment method.

**Figure 5:**
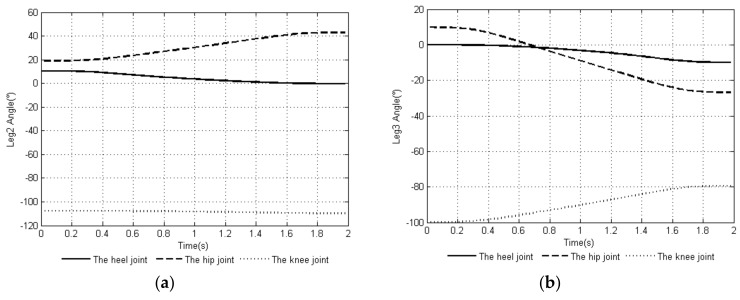

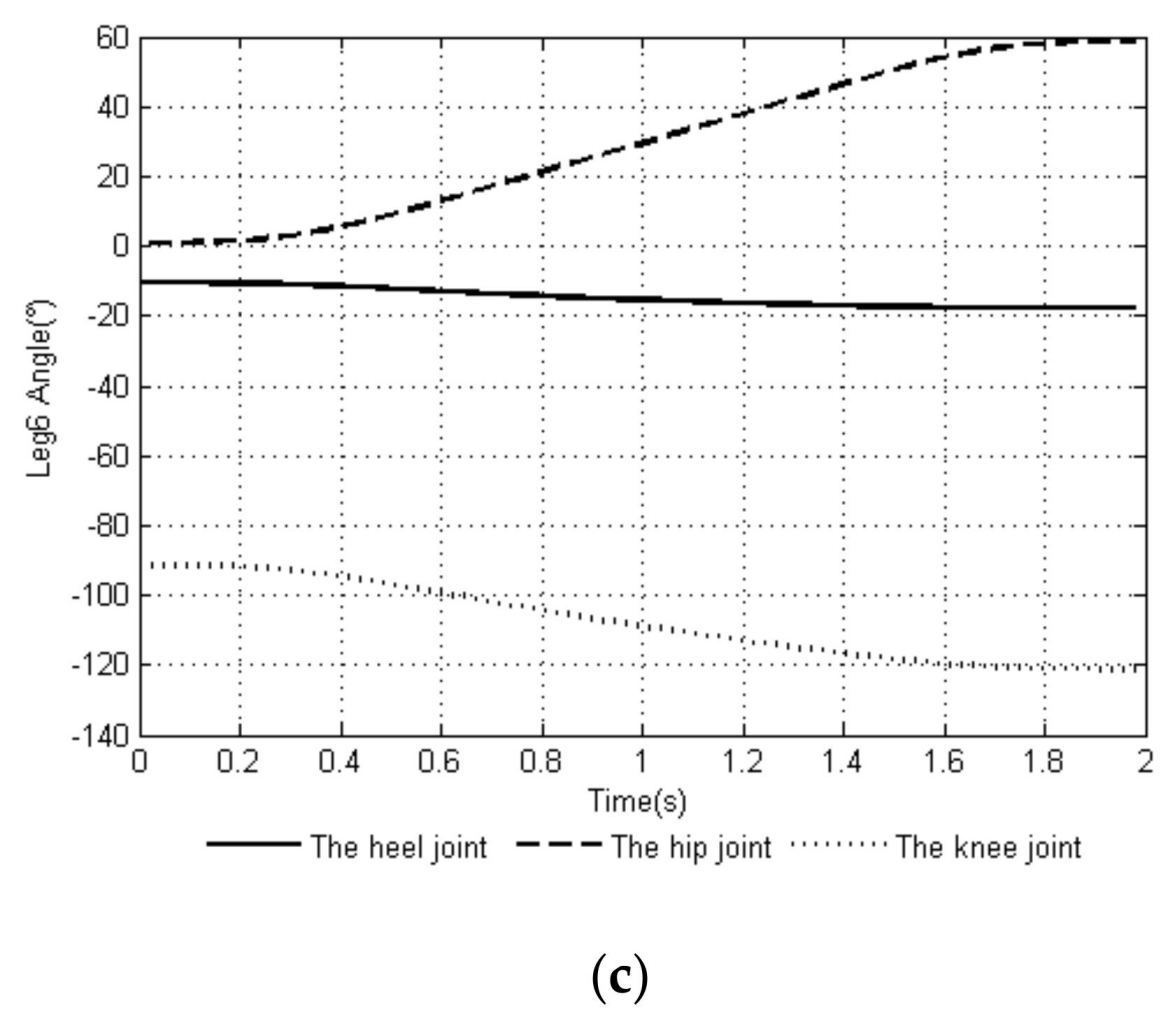
Time-curve of the joint angle of the support legs: (**a**) joint angle change of leg 2, (**b**) joint angle change of leg 3, and (**c**) joint angle change of leg 6.

**Figure 6:**
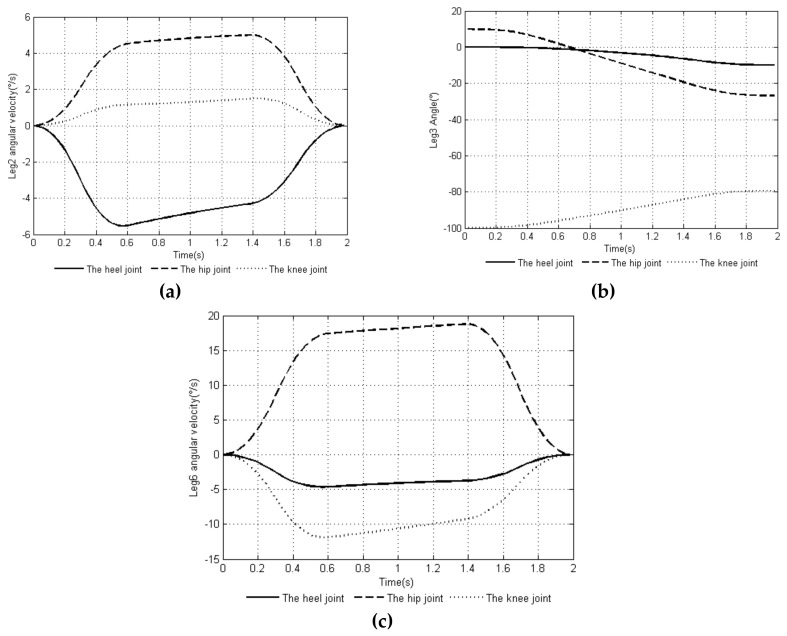
Time-curve of angular velocity of the joints of the support legs: (**a**) joint angular velocity change of leg 2, (**b**) joint angular velocity change of leg 3, and (**c**) joint angular velocity change of leg 6.

Through MATLAB, a comparison experiment of the uniform speed adjustment planning method [29] commonly used in the attitude angle and the smooth adjustment method proposed in this article was performed. The attitude angular velocity curves of the two methods are shown in Figure 3a,b, and the attitude angular acceleration curves of the two methods are shown in Figure 4a,b. The curves of the changes in the leg angle and the angular velocity of the joints of the support leg when planning with the smooth adjustment method are shown in Figure 5 and Figure 6.

Comparing the attitude angular velocity curves of the two planning methods in Figure 3, the angular velocity curve of the uniform speed adjustment method was not as smooth during the entire adjustment cycle compared with the smooth adjustment method, and the speed changed rapidly at the initial and end stages of the adjustment cycle. It can be seen in Figure 4 that the smooth adjustment method had continuous acceleration curves and no abrupt changes throughout the adjustment period, thus satisfying the constraint index of the smooth posture adjustment of (10). However, the acceleration curve of the uniform speed adjustment method was discontinuous and abrupt.

Figure 5 and Figure 6 show the joint angle and angular velocity changes of the support legs *leg2*, *leg3*, and *leg6*. It can be seen from the image that each support leg satisfied the joint angle constraint and joint angular velocity constraint conditions in Equation (10). The support leg joint angle and angular velocity curve continuously changed with time, and the steering gear rotated smoothly.

**Figure 7:**
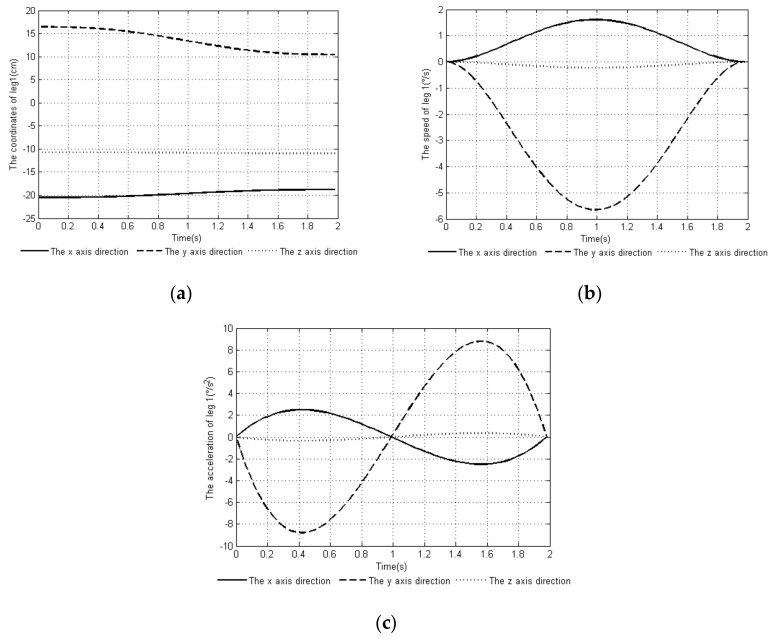
Time-curve of foot end changes with moving legs: (**a**) position change at the end of the foot, (**b**) speed change at the end of the foot, and (**c**) acceleration change at the end of the foot.

For the follower leg *leg1*, Equation (23) calculates that at the end of the posture adjustment, the center position of the contact space between the foot end and the ground is ${}^{W}P_{a}=\left[-18.8,10.46,-10.96\right]$ so point ${}^{W}P_{a}$ is taken as the landing point of the follower leg at the end time. The constraint of Equation (24) was substituted into Equation (26) to get the planning curve of the foot end of the follower leg *leg1*. The coordinates, velocity, and acceleration curves of the end points of the foot in the three directions of x, y, and z are shown in Figure 7.

## 6. Prototype Experiment

### 6.1. Experiment of the Degree of Fit Between the Target Value and the Actual Value of the Planning Curve

**Figure 8:**
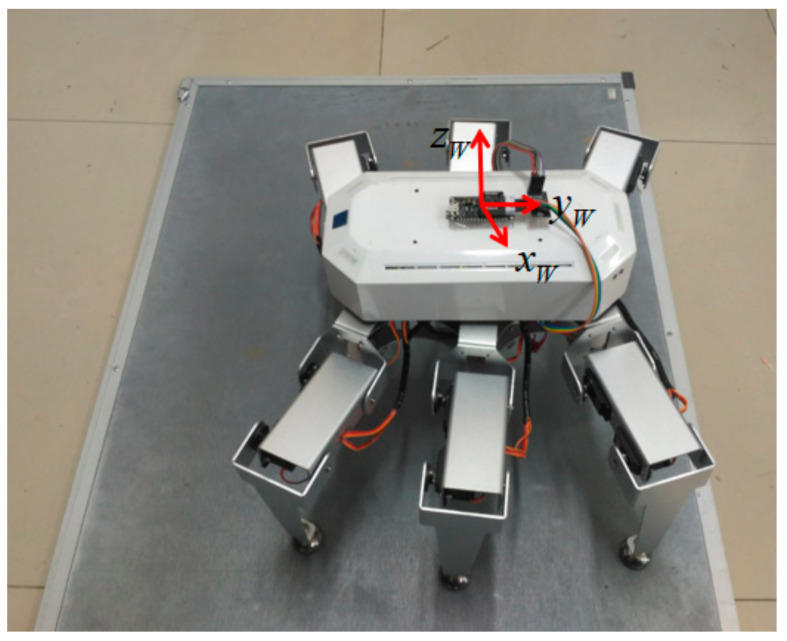
Experimental environment diagram of pose adjustment.

**Figure 9:**
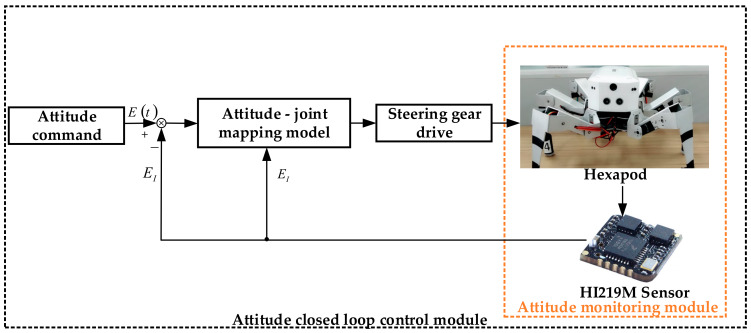
Frame diagram of closed loop control of posture of hexapod robot. Here, $E\left(t\right)$ is the real-time target attitude angle matrix. $E_{I}$ is the attitude angle matrix detected by the HI219M sensor in real time.

In order to verify the reliability of the theory, the robot is equipped with a nine-axis attitude sensor HI219M (Hipnuc inc., Beijing, China) [30] to establish an attitude monitoring environment for comparative experiments. The nine-axis gyroscope is installed in the center of the top of the robot and used as a measuring device. The attitude monitoring environment is shown in Figure 8. The sensor parameters are shown in Table 4. With the help of this sensor, the robot’s attitude angle, angular velocity, and angular acceleration data in the X, Y, and Z directions can be collected in real time, and transmitted to the host computer through the communication module for real-time drawing. The entire control block diagram during posture adjustment is shown in Figure 9.

**Figure 10:**
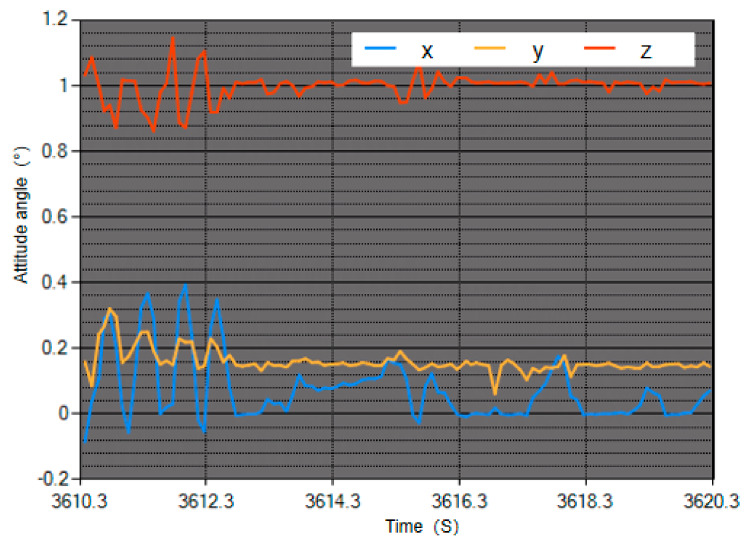
HI219M host computer software interface diagram. Here, x represents yaw angle change information, y represents pitch angle change information, and z represents roll angle change information.

The experimental process should follow the following steps:

1. Check HI219M. Since the sensor is easily affected by the geomagnetic field, it is necessary to check the accuracy of the sensor before collecting the posture. If the sensor is not checked, the sensor may be affected by the surrounding magnetic field, resulting in inaccurate measurement results. Open the interface of the sensor host and connect the module. Open the IMU interface and rotate 360° around the three coordinate axes. If a relatively complete circle appears in the drawing area, it indicates that there is no large magnetic field around the circle, so attitude measurement can be performed.
2. Confirm the experimental environment. Turn on the robot and place it under the experimental terrain to check whether the robot body has loose parts, whether the support plane is hard, and the initial posture is correct. If the experimental environment is accurate, please proceed to the next step.
3. Collect real-time images. The sensor collects the posture information of the robot to the host computer for real-time drawing during posture adjustment. Taking a random attitude angle as an example, the interface of the host computer is shown in Figure 10.

**Figure 11:**
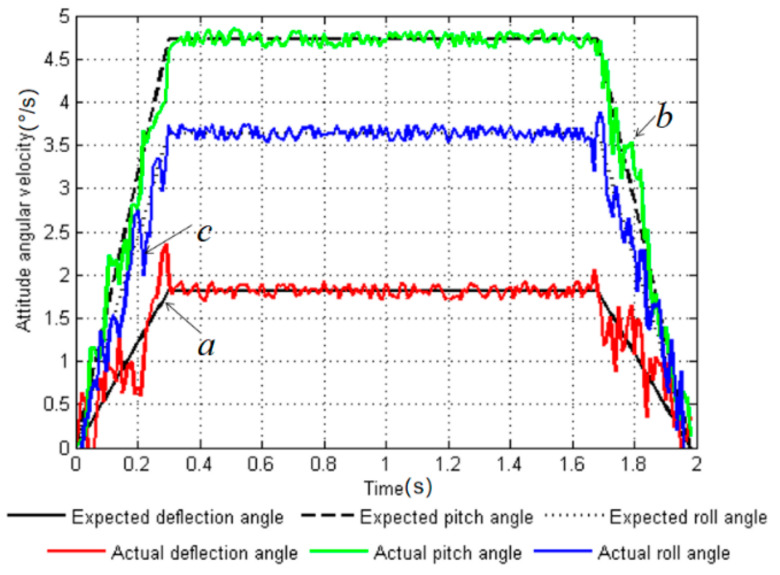
Curves of expected value and actual value of angular velocity with uniform speed adjustment method. Here, a,b and c represent the points where the robot has the largest deviation between the theoretical value and the measured value under the uniform speed adjustment method.

**Figure 12:**
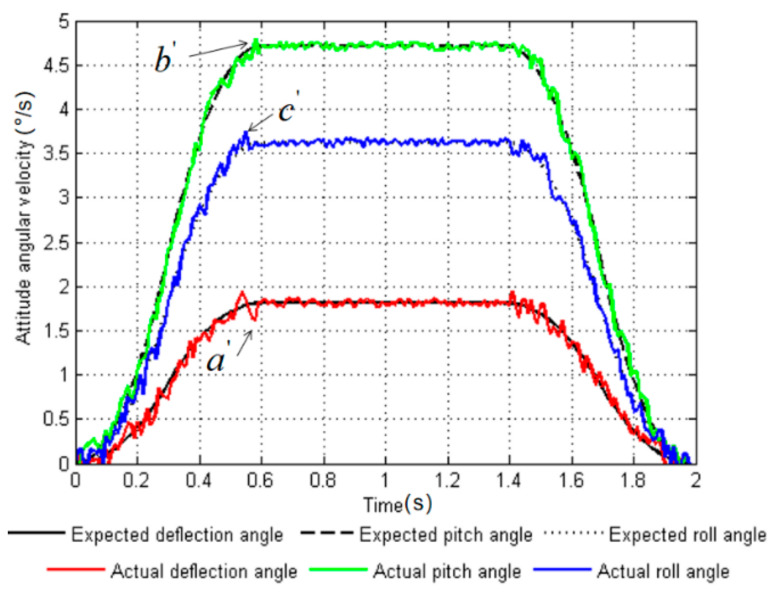
Curves of expected value and actual value of angular velocity with smooth adjustment method. $a^{\prime }$,$b^{\prime }$ and $c^{\prime }$ represent the points where the robot has the largest deviation between the theoretical value and the measured value under the smooth adjustment method.

The experimental environment was the same as the simulation environment in Section 4. The robot adjusted its attitude on three support legs (*leg2*, *leg3*, and *leg6*) on a five-degree slope. The body posture was adjusted from the initial attitude angle $E_{f}={\left[0^{{}^{\circ}},5^{{}^{\circ}},0^{{}^{\circ}}\right]}^{T}$ to the target attitude angle $E_{a}={\left[5^{{}^{\circ}},18^{{}^{\circ}},10^{{}^{\circ}}\right]}^{T}$, and the period T=1.98 s was adjusted. Taking the angular velocity information during posture adjustment as an example, the degree of fit between the target value of the angular velocity and the actual value was analyzed when using the uniform speed adjustment method (as shown in Figure 11), and the degree of fit of the target value of the angular velocity and the actual value was analyzed when using the smooth adjustment method (as shown in Figure 12). The colored lines in the figures represent the actual values collected by the sensor, and the black lines are the target values of the simulation.

It can be seen from Figure 11 that the angular velocity planned by the uniform speed adjustment method had a strong fluctuation at *t* = 0.2 s and 1.5 s, which was caused by the discontinuity in the acceleration of the uniform speed adjustment. However, the curves of the target value and the actual value for the smooth adjustment method (Figure 12) had good follow ability, and the degree of fit was higher than that of the uniform speed adjustment method. In Figure 11, the three points a, b, and c represent the points where the deviations of the actual angular velocity of the yaw angle, pitch angle, and roll angle from the target values are greatest in the uniform speed adjustment method. In Figure 12, the three points $a^{\prime }$, $b^{\prime }$, and $c^{\prime }$ are used to indicate the points where the actual angular velocity values deviate from the target values of the yaw angle, pitch angle, and roll angle in the smooth adjustment method. The theoretical and measured values of the two adjustment methods are listed in Table 5.

After calculation, the maximum deviations of the uniform speed adjustment method from the actual targets were 62.5%, 8.1%, and 30.1%, respectively. The maximum deviations of the smooth adjustment method from the actual targets in the three directions reached 5.5%, 3.2%, and 2.7%, respectively. From the analysis of the data of the representative points, it was found that the smooth adjustment method had a 57.0% increase in the degree of fit between the target angular velocity and the actual value compared with the uniform speed adjustment method. The experiment proves that by adopting the smooth adjustment method proposed in this paper, the actual value of the planned curve fits the target value well, the fluctuation is small, and the follow ability and stability are greatly improved.

### 6.2. Comparative Experiment on Stability of two Planning Methods under Load

**Figure 13:**
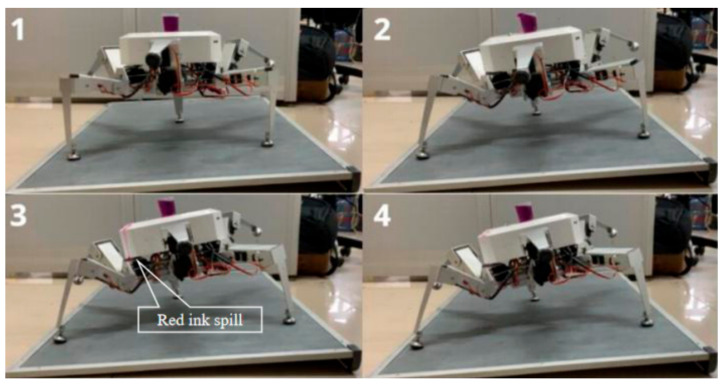
Process diagram of uniform speed adjustment method.

**Figure 14:**
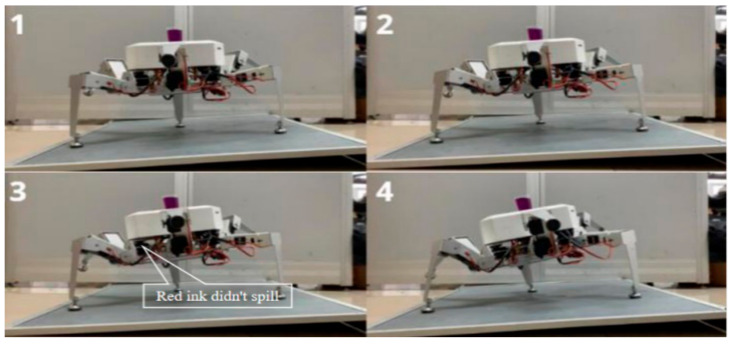
Process diagram of smooth adjustment method.

In order to more intuitively show the comparison of the two planning methods under load conditions, the robot was equipped with red ink for comparison experiments. In this experimental environment, two attitude planning methods were compared using the robot’s pitch angle in one direction. The robot adjusted its attitude on a three-legged support on a 10° slope. The attitude angle changed from the initial attitude angle $E_{f}={\left[0^{{}^{\circ}},10^{{}^{\circ}},0^{{}^{\circ}}\right]}^{T}$ to the target attitude angle $E_{a}={\left[0^{{}^{\circ}},40^{{}^{\circ}},0^{{}^{\circ}}\right]}^{T}$, and the adjustment period was T=2.5 s. A measuring cup with a capacity of 30 mL filled with red ink was fixed at the central position of the robot, and the liquid level of the measuring cup had no fluctuation in the initial state. The process of the uniform speed adjustment method in the experimental process is shown in Figure 13, and the process of the smooth adjustment method is shown in Figure 14.

As shown in Figure 13, due to the discontinuity of attitude angular velocity and angular acceleration of the uniform speed adjustment method, the sudden change of inertial force in the process of posture adjustment caused the body to shake, and the red ink in the measuring cup overflowed and dripped along the body shell to the support surface. According to the statistics, the red ink level was reduced from 30 mL to 22 mL, and the loss rate was up to 26.7%. Figure 14 shows a photograph of the state of the smooth adjustment method. As the curves of the smooth adjustment method’s attitude angular velocity and angular acceleration were continuous without mutation, the red ink level in the measuring cup changed smoothly without overflow during the whole adjustment period, and the fuselage did not shake greatly during the whole adjustment period. This adjustment method helps the robot to obtain stability.

### 6.3. Planning Experiment of Follower Leg

**Figure 15:**
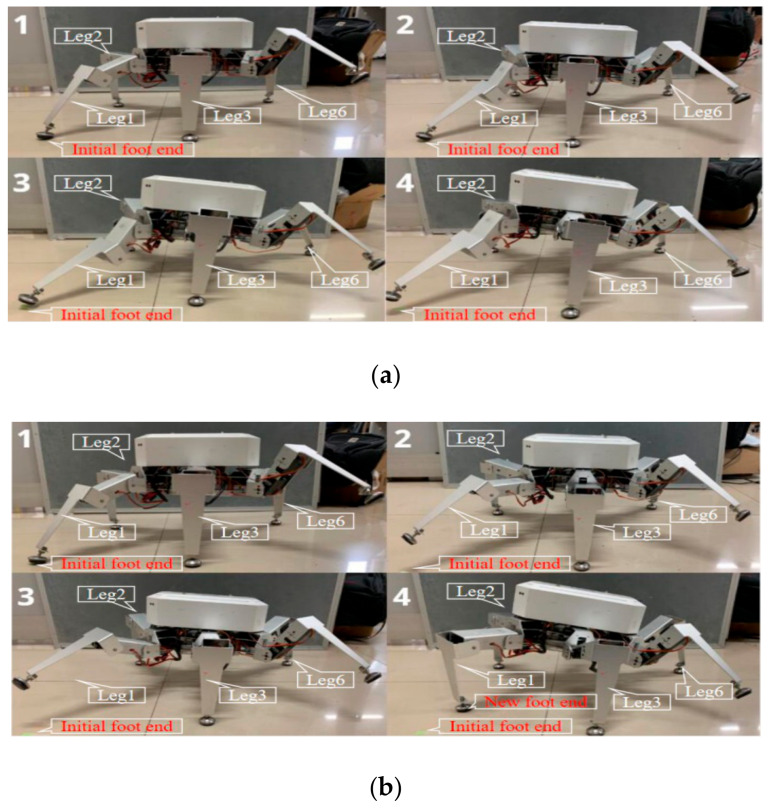
Comparison chart of planning of follower leg. (**a**) Screenshot of unplanned follower leg track. (**b**) Screenshot of the completed tracking leg track.

In order to verify the effect of the planning of follower leg on the body, a comparative experiment was performed with the robot’s pitch and unidirectional attitude changes. In planar terrain, the attitude angle was adjusted from the initial attitude angle $E_{f}={\left[0^{{}^{\circ}},0^{{}^{\circ}},0^{{}^{\circ}}\right]}^{T}$ to the target attitude angle $E_{a}={\left[0^{{}^{\circ}},30^{{}^{\circ}},0^{{}^{\circ}}\right]}^{T}$, and the period T=2.8 s was adjusted. The robot had four legs—*leg1*, *leg2*, *leg3*, and *leg6*. When considering the planning of follower leg, the robot judged that *leg2*, *leg3*, and *leg6* were the support legs, and *leg1* was the follower leg. The projection of the center of mass on the water surface during the adjustment process was within the supporting triangle. A photograph of the adjustment process is shown in Figure 15.

From the analysis of the two sets of images in Figure 15, it can be seen that when the robot did not carry out planning of follower leg, support *leg1* reached the upper and lower limits of the knee angle during postural adjustment. Therefore, if planning of follower leg is not done, the steering gear will be seriously damaged, and the robot’s motion will be affected. When the robot carried out planning of follower leg, the follower leg moved to a new landing point according to the planning curve of Equation (25), and the posture was adjusted smoothly in cooperation.

In this section, the deviations between the target values and the actual values of the planned curves of the two adjustment methods were compared with the attitude sensor experiments carried out on the prototype of the six-legged robot. The stability of the body during posture adjustment was compared through two adjustment methods equipped with red ink load experiments, which proved that the smooth adjustment method is more stable. Through the planning of follower leg experiment, it was proved that the planned follower leg can be synchronized with the posture adjustment and will not affect the body’s movement trend. In summary, when the robot adjusts its attitude, the smooth adjustment method can maintain the robot’s good stability and improve the terrain adaptability of the robot.

## 7. Conclusions

The attitude transformation of a hexapod robot on terrain is of great significance to the efficiency and stability of robot operation. Aiming to solve the problem that the hexapod robot is prone to shaking during posture adjustment, this paper proposed a smooth posture adjustment strategy. The influence of the support leg on the robot during posture adjustment was analyzed. A smooth adjustment method was developed and compared with the traditional uniform speed adjustment method by simulation and experiment. Through simulation and experiments, the degree of fit between the target value and the actual value of the two adjustment methods and the stability of the fuselage under the load state were verified. The experiments showed that the target value and the actual value of the smooth adjustment method had a higher degree of fit. The body shook less during adjustment, which is beneficial to the robot’s ability to adapt to terrain.

The innovations of this paper are as follows: First, all the support legs of the robot’s posture adjustment were divided into two types, which were separately planned and completed synchronously. Second, aiming at the problem of the robot body shaking when adjusting the attitude of the robot, a smooth adjustment method was designed to continuously change the attitude angular acceleration starting from and ending at zero. This study solved the problem of the instability of the fuselage when the robot changes its attitude and provided an important reference for the multi-footed robot to improve the terrain adaptability.

The follow-up work will study the smooth adjustment of the robot’s posture and position so that the robot can make smooth adjustments in a manner closer to that of living things. Furthermore, the robot’s foot end uses a universal foot structure, and the rigid contact between the foot end and the terrain will cause the body to shake to some extent. In the future, the foot end structure will be improved by equipping the robot with a flexible foot end to further reduce the foot impact and further improve the smooth running of the robot.

## Figures and Tables

**Table 1:** The parameters of the robot.

Structural Parameter Name	Symbolic Representation	Structural Parameter Name	Symbolic Representation
Base length	$l_{1}$	Heel joint angle	$\theta_{i1}$
Femoral length	$l_{2}$	Hip joint angle	$\theta_{i2}$
Tibia length	$l_{3}$	Knee joint angle	$\theta_{i3}$
Body pitch	L	Body width	$\lambda_{1},\;\lambda_{2}$
The *i*-th legs	legi(i=1,2,…,6)		

**Table 2:** Specific parameters of hexapod robot.

Structural Name	Value	Structural Name	Value Range
Base length ($l_{1}$)	6 cm	Heel joint angle ($\theta_{i1}$)	−30°–30°
Femoral length ($l_{2}$)	7 cm	Hip joint angle ($\theta_{i2}$)	−90°–90°
Tibia length ($l_{3}$)	13 cm	Knee joint angle ($\theta_{i3}$)	−150°–0°
Body pitch (L)	10 cm	Joint angular velocity range ($V_{e}$)	(5°/s, 20°/s, 15°/s)*^T^*
Body width ($\lambda_{1},\lambda_{2}$)	4 cm, 3 cm		

**Table 3:** Experiment parameters.

	Parameters	Value
**Measurements**	*Leg 1* initial angle ($\theta_{1}$)	(19.4°, −5.0°, −54.2°)*^T^*
	*Leg 2* initial angle ($\theta_{2}$)	(10°, 20°, −108°)*^T^*
	*Leg 3* initial angle ($\theta_{3}$)	(0°, 10°, −100°)*^T^*
	*Leg 6* initial angle ($\theta_{6}$)	(−10°, 2°, −92°)*^T^*
**Calculated**	*Leg 1* Foot End Position (${}^{W}P_{1}$)	(−20.5 cm, 16.43 cm, −10.72 cm]*^T^*
	*Leg 2* Foot End Position (${}^{W}P_{2}$)	(14.7 cm, 12.35 cm, −10.72 cm)*^T^*
	*Leg 3* Foot End Position (${}^{W}P_{3}$)	(−14.4 cm, 0 cm, −11.8 cm)*^T^*
	*Leg 6* Foot End Position (${}^{W}P_{6}$)	(14.7 cm, −12.35 cm, −12.88 cm)*^T^*

**Table 4:** Sensor parameters.

Size	12 × 12 mm
Data output rate	0–1000 Hz
Resolution	±0.01 °~±0.1 °
Serial output rate	115,200 bit/s

**Table 5:** Statistical table of theoretical and measured values.

Adjustment Method	Uniform Speed Adjustment Method			Smooth Adjustment Method		
	a	b	c	$a^{\prime }$	$b^{\prime }$	$c^{\prime }$
**Theoretical value n (°/s)**	1.52	3.33	3.05	1.75	4.75	3.65
**Measurements m (°/s)**	2.47	3.60	2.12	1.65	4.60	3.75
**Deviation λ (%)**	62.5	8.10	30.5	5.50	3.20	2.70
